# Enhanced passive surveillance of influenza vaccination in England, 2016−2017– an observational study using an adverse events reporting card

**DOI:** 10.1080/21645515.2019.1565258

**Published:** 2019-03-20

**Authors:** Simon de Lusignan, Filipa Ferreira, Silvia Damaso, Rachel Byford, Sameera Pathirannehelage, Anne Yeakey, Ivelina Yonova, Anne Schuind, Gael Dos Santos

**Affiliations:** a Department of Clinical & Experimental Medicine, University of Surrey, Guildford, UK; b Biostatistics EPI, GSK, Wavre, Belgium; c Clinical Safety & Pharmacovigilance, GSK, Rockville, MD, USA; d Clinical R&D, GSK, Rockville, MD, USA; e Clinical R&D, GSK, Wavre, Belgium

**Keywords:** [MeSH]: General practice, medical record systems, computerized, pharmacovigilance, records as topic, drug-related side effects and adverse reactions, adverse drug reaction reporting systems, adverse effects, influenza vaccines, influenza, human

## Abstract

Influenza is a major public health burden, mainly prevented by vaccination. Recommendations on influenza vaccine composition are updated annually and constant benefit-risk monitoring is therefore needed. We conducted near-real-time enhanced passive surveillance (EPS) for the influenza vaccine, *Fluarix Tetra*, according to European Medicines Agency guidance, in 10 volunteer general practices in England using *Fluarix Tetra* as their principal influenza vaccine brand, from 1-Sep to 30-Nov-2016. The EPS method used a combination of routinely collected data from electronic health records (EHR) and a customized adverse events reporting card (AERC) distributed to participants vaccinated with *Fluarix Tetra*. For participants vaccinated with a different influenza vaccine, data were derived exclusively from the EHR. We reported weekly and cumulative incidence of pre-defined adverse events of interest (AEI) occurring within 7 days post-vaccination, adjusted for clustering effect. Of the 97,754 eligible participants, 19,334 (19.8%) received influenza vaccination, of whom 13,861 (71.7%) received *Fluarix Tetra*. A total of 1,049 participants receiving *Fluarix Tetra* reported AEIs; 703 (67%) used the AERC (adjusted cumulative incidence rate 4.96% [95% CI: 3.92−6.25]). Analysis by individual pre-specified AEI categories identified no safety signal for *Fluarix Tetra*. A total of 62 individuals reported an AEI with a known brand of non-GSK influenza vaccine and 54 with an unknown brand (adjusted cumulative incidence rate 2.59% [1.93−3.47] and 1.77% [1.42−2.20], respectively). In conclusion, the study identified no safety signal for *Fluarix Tetra* and showed that the AERC was a useful tool that complemented routine pharmacovigilance by allowing more comprehensive capture of AEIs.

**Figure:**
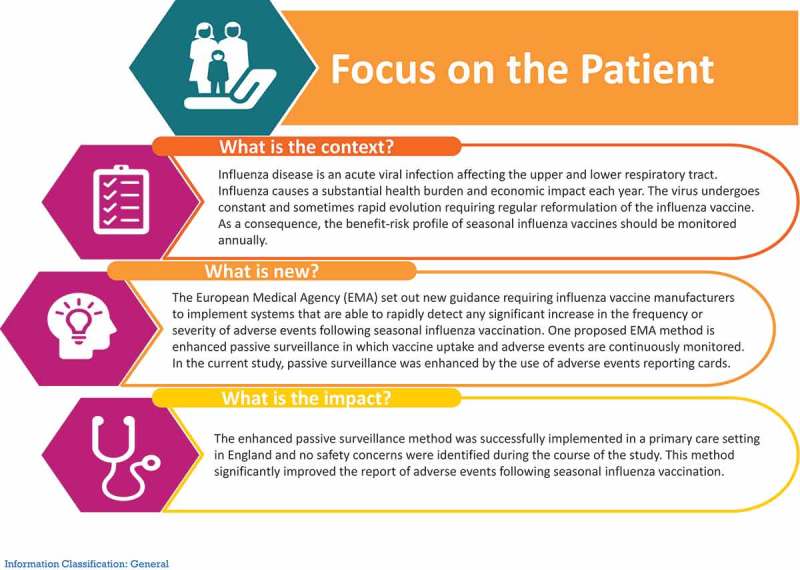

## Introduction

Influenza is a major public health burden resulting in significant clinical and economic impact each year. It is estimated that 290,000–650,000 seasonal influenza-associated respiratory deaths occur globally and between 15,000 and 70,000 deaths in the European Union and European Economic Area Member States each year.^1-3^ According to the World Health Organization (WHO), the most effective way to prevent the disease is vaccination, although other personal protective measures should also be adopted, like good hand and respiratory hygiene, avoiding contact with sick people and early self-isolation of those with influenza symptoms.^4^ Due to frequent genetic and antigenic changes in influenza viruses, seasonal vaccines are regularly reformulated based on annual WHO recommendations on the composition of influenza virus vaccines.^5^ Therefore, there is a need for constant benefit-risk monitoring of these vaccines.

Approaches to pharmacovigilance are constantly evolving to ensure that medicines and vaccines are safe and public confidence in them is maintained. A fall in public confidence can lead to public health issues; for example, the withdrawal of two batches of a specific brand of influenza vaccine in Italy during the 2014–2015 season resulted in a decline in influenza vaccine uptake.^6,7^ Furthermore, concerns about adverse events after vaccination with pandemic influenza A/H1N1 vaccine suggested that surveillance systems in place at the time may have been inadequate.^8^

The role of the European Medicines Agency (EMA) includes supervision of pharmacovigilance activities by pharmaceutical companies.^9^ The European regulatory requirement for manufacturers of seasonal influenza vaccines to conduct annual small scale clinical trials was withdrawn in 2015. Instead, the EMA has set out new guidance for influenza vaccine monitoring,^10^ which should be seen in the context of the EMA’s wider approach to good pharmacovigilance practice.^11,12^ The goal of the new guidance is to be able to rapidly detect, in near-real-time early in the season, any significant increase in the frequency or severity of pre-defined adverse events of interest (AEIs).^10^ The EMA has suggested three options for vaccine manufacturers to monitor AEIs following vaccination: (1) active surveillance, using existing methods of post authorization surveillance; (2) enhanced passive surveillance (EPS) in which vaccine usage is rapidly estimated and additional steps are taken to facilitate passive reporting of AEIs; and (3) data mining or other use of electronic health records (EHRs).^10-12^

General practice in England is an appropriate setting to implement EPS as specified by the EMA. It has a registration-based list system (one individual registered with one general practitioner [GP]) and has been highly computerized since 2004, with all key data coded.^13^ Data extracted from these systems are widely used in research.^14^ General practices are largely independent professional partnerships that make their own decision about which brand of influenza vaccine to purchase prior to the start of each influenza season. Practices administer influenza vaccines to recommended groups, starting in September of each year.^15^ For the 2017−2018 influenza season, Public Health England reported uptake levels of 70% or higher in the 65 years and over age group, while an uptake of 50% was reported for individuals aged 6 months to under 65 years in clinical at-risk groups;^16^ almost all individuals received the vaccine at a general practice, whilst approximately 10% were vaccinated at community pharmacies, school or work.^16^

The present study was conducted to fulfil EMA pharmacovigilance requirements for *Fluarix Tetra* (GSK), an inactivated quadrivalent influenza vaccine. Our primary objective was to estimate on a weekly basis the crude and cumulative incidence rate of AEIs within 7 days following vaccination with a seasonal influenza vaccine. We employed an EPS method which used a combination of data routinely collected via EHR, together with data collected via an adverse events reporting card (AERC) to maximize the likelihood of capturing adverse events experienced by vaccinees receiving GSK’s *Fluarix Tetra*. Participants who received a non-GSK influenza vaccine were followed up using EHR systems only; they were not issued with an AERC. We have previously shown the feasibility of this method in general practice in England.^17,18^

## Results

The total registered population of the 10 practices was 97,801 individuals. Of these, 97,754 had not opted out of data sharing, had a valid National Health Service (NHS) number, and their age and gender recorded in their EHR, and were therefore eligible to participate in the study.

### Influenza vaccine exposure

**Figure 1.**
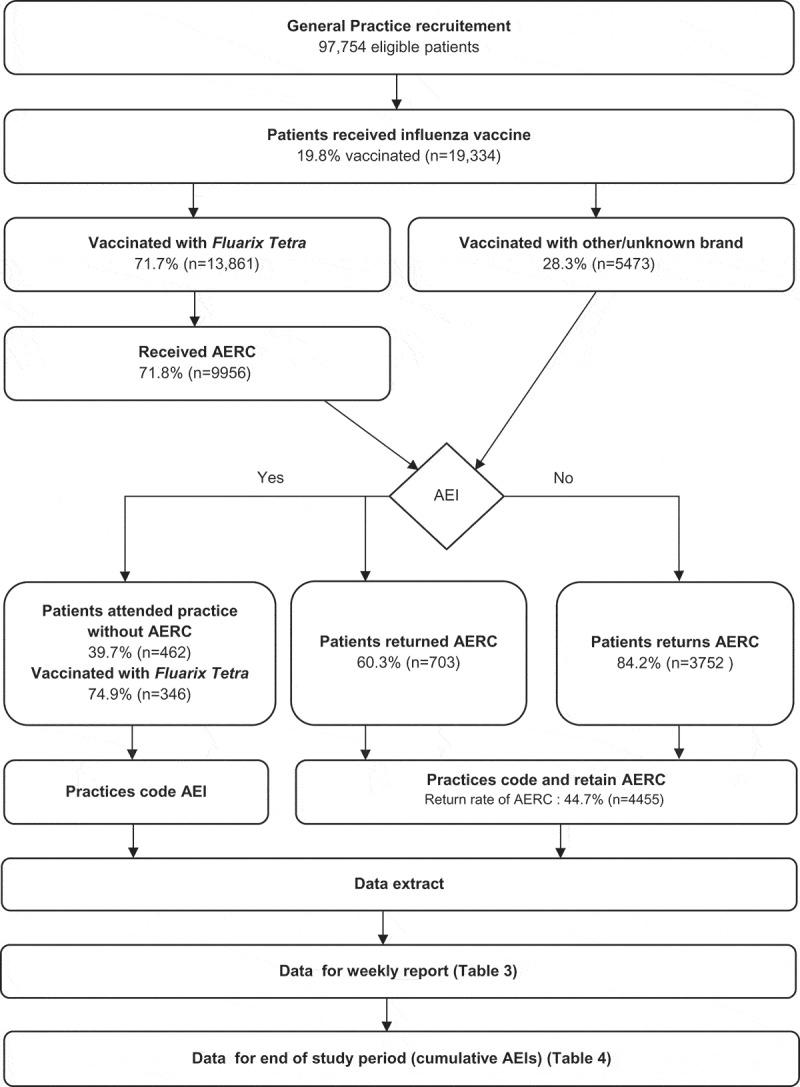
Flow diagram of study. AEI: adverse events of interest; AERC: adverse events reporting card

The study vaccination period covered 1 September to 30 November 2016 and the follow-up period captured AEIs from week 37 to week 48. A total of 19,334 out of 97,754 (19.8%) individuals were vaccinated against influenza (Tables 1 and 2). Most vaccinees (71.7%) received *Fluarix Tetra* (Figure 1). Information on vaccination data (vaccine administration date and batch number) in the EHR was >99% complete for *Fluarix Tetra* and non-GSK influenza vaccine brands (Supplement Table 1). The non-GSK vaccines administered were LAIV manufactured by Astra Zeneca (quadrivalent) and inactivated vaccines manufactured by Sanofi Pasteur Europe (trivalent); Seqirus Vaccines Limited (trivalent); and Abbot Biologicals (trivalent).

**Table 1.** Summary of demographic characteristics by vaccine group.

		*Fluarix Tetra* N = 13,861		Non-GSK N = 2295		Unknown brand N = 3178		All vaccinated N = 19,334	
Characteristics	Parameters or Categories	Value or n	%	Value or n	%	Value or n	%	Value or n	%
Age* (years)	Mean	66		24		57		60	
	SD	16.2		29.7		24.9		24.1	
	Median	70		5		66		68	
	Minimum	0		1		1		0	
	Maximum	107		102		97		107	
Gender	Female	7661	55.3	1159	50.5	1808	56.9	10,628	55.0
Ethnicity	Asian	327	2.4	244	10.6	123	3.9	694	3.6
	Black	275	2.0	334	14.6	133	4.2	742	3.8
	White	9581	69.1	906	39.5	1506	47.4	11,993	62.0
	Mixed	39	0.3	64	2.8	10	0.3	113	0.6
	Other	36	0.3	19	0.8	5	0.2	60	0.3
	Missing	3603	26.0	728	31.7	1401	44.1	5732	29.6
Index of Multiple Deprivation (IMD) (0 = least and 100 = most deprived)	N’	13,236		2232		2545		18,013	
	Mean	16.0		21.8		20.0		17.3	
	SD	12.2		13.2		13.5		12.7	
	Median	11.9		22.4		16.4		13.3	
	Minimum	1.3		1.3		1.4		1.3	
	Maximum	66.3		66.3		70.5		70.5	
	Missing	625		63		633		1321	

*Age at influenza vaccination.

N: number of participants.

N’: number of participants with available data.

n/%: number/percentage of participants in a given category; SD: standard deviation.

**Table 2.** Summary of age category and risk group by vaccine group.

			*Fluarix Tetra*			Non-GSK			Unknown brand			All vaccinated	
Group	Categories	N	n	%	%c	n	%	%c	n	%	%c	n’	%
Age	Any age	97,754	13,861	14.2	71.7	2295	2.3	11.9	3178	3.3	16.4	19,334	19.8
	6 months - 5 years	6826	16	0.2	1.2	1168	17.1	87.1	157	2.3	11.7	1341	19.6
	6–12 years	8019	24	0.3	4.2	275	3.4	48.3	270	3.4	47.5	569	7.1
	13–17 years	5714	54	0.9	34.8	83	1.5	53.5	18	0.3	11.6	155	2.7
	18–65 years	61,088	4794	7.8	76.9	370	0.6	5.9	1070	1.8	17.2	6234	10.2
	>65 years	16,107	8973	55.7	81.3	399	2.5	3.6	1663	10.3	15.1	11,035	68.5
Risk group	Any risk group	39,222	12,591	32.1	75.2	1639	4.2	9.8	2512	6.4	15.0	16,742	42.7
	Asthma	12,319	3048	24.7	75.7	361	2.9	9.0	614	5.0	15.3	4023	32.7
	Chronic respiratory disease	2308	1256	54.4	78.0	76	3.3	4.7	279	12.1	17.3	1611	69.8
	Chronic heart disease	6028	3228	53.6	81.4	178	3.0	4.5	561	9.3	14.2	3967	65.8
	Chronic kidney disease	3873	2317	59.8	84.5	77	2.0	2.8	348	9.0	12.7	2742	70.8
	Chronic liver disease	5693	1896	33.3	79.3	121	2.1	5.1	374	6.6	15.6	2391	42.0
	Diabetes	2483	1235	49.7	73.4	104	4.2	6.2	344	13.9	20.4	1683	67.8
	Immunosuppression (includes relevant cancer treatment)	559	251	44.9	78.0	24	4.3	7.5	47	8.4	14.6	322	57.6
	Chronic neurological disease	4116	2000	48.6	82.7	131	3.2	5.4	287	7.0	11.9	2418	58.8
	Asplenia	757	213	28.1	77.5	33	4.4	12.0	29	3.8	10.5	275	36.3
	Pregnancy	2110	411	19.5	80.9	31	1.5	6.1	66	3.1	13.0	508	24.1
	Under 4 years old	4312	5	0.1	0.6	732	17.0	93.1	49	1.1	6.2	786	18.3
	Over 65 years old	16,084	8973	55.8	81.3	399	2.5	3.6	1663	10.4	15.1	11,035	68.7
	Not at risk	58,532	1270	2.2	49.0	656	1.1	25.3	666	1.1	25.7	2592	4.4

N = number of participants in a given category.

n = number of participants in the given category who received the specified seasonal influenza vaccine.

n’ = number of participants in the given category who received any seasonal influenza vaccine.

% = (n/N) * 100 for a specified vaccine brand or (n’/N) * 100 for any vaccine brand.

%c = (n/n’) * 100.

Generally, GPs used *Fluarix Tetra* preferentially for older people: the mean age for people receiving *Fluarix Tetra* was 66 years versus 24 years for people receiving non-GSK brands (Table 1). Considering all age groups, the overall uptake of influenza vaccine was 19.8%. In the >65 years age group, 68.5% received any influenza vaccine, of whom 81.3% received *Fluarix Tetra* (Table 2). Among people with any high-risk condition, uptake of any influenza vaccine was 42.7%, of whom 75.2% received *Fluarix Tetra* (Table 2). Uptake of influenza vaccine was relatively low in pregnant women and children <4 years of age (24.1% and 18.3% overall, respectively; Table 2).

### Adverse events of interest reported for Fluarix Tetra

Of the 13,861 participants who were immunized with *Fluarix Tetra*, 9956 (71.8%) received an AERC, of whom 4455 (44.7%) returned it (note that individuals who were immunized with another brand were not given an AERC) (Figure 1). A total of 1049 people receiving *Fluarix Tetra* reported AEIs overall; 67.0% (n = 703) of these were reported using the AERC.

#### Events reported for Fluarix Tetra via AERC

**Figure 2.**
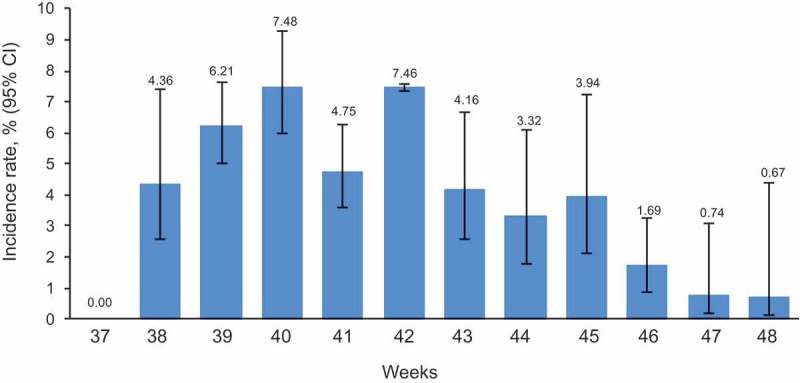
Weekly incidence rate of any AEIs reported via AERC within 7 days post-vaccination in participants receiving *Fluarix Tetra.* The figure shows the percentage of participants reporting an AEI at least once on the AERC estimated from logistic GEE models adjusted for clustering effect of general practices, with upper and lower limits of the 95% CI based on the robust variance estimate AEI: adverse event of interest; AERC: adverse event reporting card; 95% CI: 95% confidence interval; GEE: generalized estimating equation; LL: lower limit; UL: upper limit

Weekly incidence rates via AERC were reported throughout the follow-up period (weeks 37 to 48). These varied from 0.67% (n = 2) at week 48 to 7.48% (n = 164) at week 40. Rates peaked between weeks 39−42, followed by a steady decrease throughout the remainder of the study (Figure 2; Supplement Table 2).

**Figure 3.**
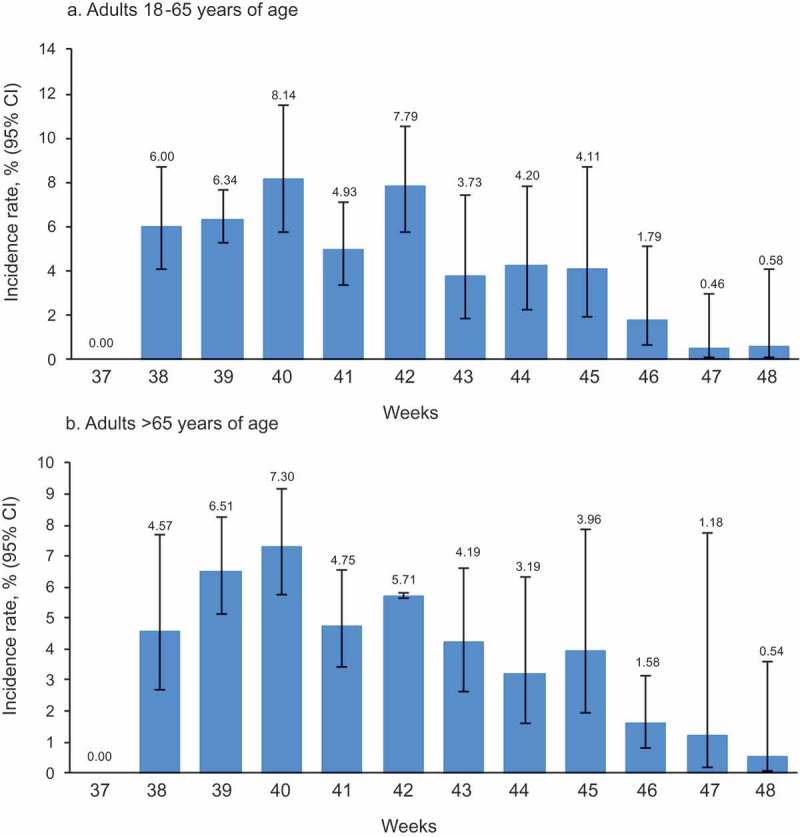
Weekly incidence rate of any AEIs reported via AERC within 7 days post-vaccination according to age group in adult participants receiving *Fluarix Tetra.* The figure shows the percentage of participants reporting an AEI at least once on the AERC estimated from logistic GEE models adjusted for clustering effect of general practices, with upper and lower limits of the 95% CI based on the robust variance estimate AEI: adverse event of interest; AERC: adverse event reporting card; 95% CI: 95% confidence interval; GEE: generalized estimating equation; LL: lower limit; UL: upper limit

Across the follow-up period, AEIs were reported by one child in the 13−17 years age group, 238 adults aged 18−65 years, and 464 adults aged >65 years. Rates for adults aged 18–65 and over 65s peaked in weeks 39–42 and then declined (Figure 3(a,b)).

The cumulative incidence rate of AEIs reported using the AERC for *Fluarix Tetra* during the follow-up period was 4.96% (95% confidence interval [CI]: 3.92−6.25) (Supplement Table 2). The cumulative incidence rates were 5.01% (95% CI: 3.77−6.62) in adults 18−65 years of age and 5.01% (95% CI: 4.02−6.23) in adults >65 years of age (Supplement Table 3).

**Table 3.** Weekly incidence rates of any AEI reported on the EHR or by AERC within 7 days post-vaccination by vaccine group.

	*Fluarix Tetra*					Non-GSK					Unknown brand					All vaccinated				
				95% CI					95% CI					95% CI					95% CI	
Week	N	n	%	LL	UL	N	n	%	LL	UL	N	n	%	LL	UL	N	n	%	LL	UL
37	6	0	-	-	-	0	0	-	-	-	88	0	-	-	-	94	0	-	-	-
38	1269	87	6.11	4.10	9.01	312	4	1.29	1.29	1.30	252	7	2.04	0.74	5.51	1833	98	4.64	2.94	7.25
39	2626	227	8.48	7.82	9.19	169	10	6.20	6.18	6.23	430	3	0.70	0.20	2.39	3225	240	7.43	6.74	8.18
40	2146	218	10.15	8.77	11.72	325	11	3.61	3.58	3.63	357	4	1.22	0.33	4.33	2828	233	8.12	6.78	9.71
41	2458	172	7.46	5.94	9.34	334	5	1.46	0.41	5.01	424	7	1.71	0.98	2.98	3216	184	5.79	4.55	7.35
42	1362	118	8.64	8.53	8.76	239	10	4.41	2.70	7.11	432	8	2.46	2.39	2.53	2033	136	6.49	5.31	7.90
43	738	57	7.58	7.01	8.19	140	4	2.53	0.82	7.58	356	10	2.77	1.15	6.54	1234	71	6.01	4.68	7.68
44	769	52	6.60	4.93	8.79	179	6	3.22	1.66	6.17	204	4	1.81	0.57	5.59	1152	62	5.28	3.94	7.04
45	704	54	7.81	5.57	10.86	113	2	1.97	0.71	5.36	242	4	1.22	1.18	1.27	1059	60	5.70	4.17	7.75
46	945	37	5.00	3.72	6.69	272	6	4.21	1.68	10.16	141	2	1.54	0.59	3.94	1358	45	4.49	3.43	5.87
47	474	14	3.11	1.71	5.62	132	2	1.74	0.41	6.99	149	3	2.85	0.72	10.6	755	19	2.65	1.50	4.62
48	364	13	3.54	2.48	5.03	80	2	2.07	0.35	11.15	103	2	2.30	0.94	5.52	547	17	3.16	2.15	4.61

N: number of participants vaccinated.

n: number of participants reporting an AEI at least once.

%: percentage of participants reporting an AEI at least once estimated from logistic GEE models adjusted for clustering effect of general practices, with upper and lower limits of the 95% CI based on the robust variance estimate.

AEI: adverse event of interest; AERC: adverse event reporting card; EHR: electronic health record; GEE: generalized estimating equation; LL: lower limit; UL: upper limit; 95% CI: 95% confidence interval.

Recruitment into the study by GP practices may have created a clustering effect, because participants in the same practice tend to share certain demographic and social characteristics. We therefore calculated an intra-cluster correlation coefficient (ICC) to measure the clustering effect between general practices. Importantly, the ICC was low (0.0053), indicating homogeneity in reporting rates of AEIs via AERC between practices.

#### Total events reported for Fluarix Tetra including EHR and AERC data

The patterns of reported AEIs for both EHR and AERC were similar to those reported using just AERC. The age of study participants reporting AEIs following vaccination with *Fluarix Tetra* using AERC and EHR data was distributed as follows: two children in the 6 months to 5 years group, three children in the 6−12 years group, one child in the 13−17 years group, 384 adults in the 18−65 years group, and 659 adults in the >65 years group.

The weekly incidence rate of AEIs during the follow-up period (weeks 37 to 48) reported via AERC and EHR for *Fluarix Tetra* varied from 3.11% (n = 14) at week 47 to 10.15% (n = 218) at week 40, peaking between weeks 39−42 (Table 3). The overall cumulative rate of AEIs for *Fluarix Tetra* during the follow-up period was 7.60% (95% CI: 6.43−8.96; n = 1,049) (Table 4).

**Table 4.** Cumulative incidence rates of any AEI reported on the EHR or by AERC within 7 days post- vaccination by vaccine group.

	*Fluarix Tetra*					Non-GSK					Unknown brand					All vaccinated				
				95% CI					95% CI					95% CI					95% CI	
Week	N	n	%	LL	UL	N	n	%	LL	UL	N	n	%	LL	UL	N	n	%	LL	UL
37	6	0	-	-	-	0	0	-	-	-	88	0	-	-	-	94	0	-	-	-
38	1275	87	6.04	4.04	8.94	312	4	1.29	1.29	1.30	340	7	1.56	0.53	4.52	1927	98	4.41	2.77	6.97
39	3901	314	8.03	7.00	9.19	481	14	2.86	2.86	2.87	770	10	0.86	0.25	2.89	5152	338	6.49	5.43	7.74
40	6047	532	8.85	7.73	10.12	806	25	3.13	3.10	3.15	1127	14	1.11	0.47	2.61	7980	571	7.14	6.03	8.43
41	8505	704	8.33	6.95	9.96	1140	30	2.82	2.39	3.32	1551	21	1.35	0.80	2.28	11,196	755	6.71	5.54	8.11
42	9867	822	8.35	7.09	9.81	1379	40	3.28	3.15	3.42	1983	29	1.46	0.98	2.16	13,229	891	6.65	5.58	7.91
43	10,605	879	8.32	7.13	9.69	1519	44	2.68	1.89	3.79	2339	39	1.64	1.17	2.30	14,463	962	6.62	5.60	7.81
44	11,374	931	8.20	7.03	9.53	1698	50	3.33	3.17	3.50	2543	43	1.58	1.06	2.35	15,615	1024	6.51	5.49	7.69
45	12,078	985	8.16	7.01	9.47	1811	52	3.29	3.10	3.48	2785	47	1.61	1.14	2.28	16,674	1084	6.45	5.45	7.60
46	13,023	1022	7.89	6.65	9.35	2083	58	2.70	2.06	3.55	2926	49	1.63	1.19	2.22	18,032	1129	6.28	5.22	7.53
47	13,497	1036	7.72	6.54	9.10	2215	60	2.59	1.94	3.45	3075	52	1.74	1.37	2.21	18,787	1148	6.13	5.13	7.32
48	13,861	1049	7.60	6.43	8.96	2295	62	2.59	1.93	3.47	3178	54	1.77	1.42	2.20	19,334	1165	6.04	5.05	7.22

N: number of participants vaccinated.

n: number of participants reporting an AEI at least once.

%: percentage of participants reporting an AEI at least once estimated from logistic GEE models adjusted for clustering effect of general practices, with upper and lower limits of the 95% CI based on the robust variance estimate.

AEI: adverse event of interest; AERC: adverse event reporting card; EHR: electronic health record; GEE: generalized estimating equation; LL: lower limit; UL: upper limit; 95% CI: 95% confidence interval.

The ICC was 0.0033, indicating homogeneity in reporting rates of AEIs recorded via the EHR and AERC for *Fluarix Tetra* between general practices.

### Adverse events of interest reported for non-GSK and unknown vaccine brands via EHR

Of note, participants who were immunized with non *Fluarix Tetra* vaccines were not provided with an AERC and therefore AEIs were captured using data routinely encoded in the EHR exclusively. A total of 62 individuals reported an AEI with a non-GSK influenza vaccine and 54 with an unknown brand of influenza vaccine. Weekly AEI incidence rates during the follow-up period ranged from 1.29% to 6.20% for non-GSK brands and from 0.70% to 2.85% for unknown vaccine brands (Table 3). The overall cumulative rate of AEIs during the follow-up period was 2.59% (95% CI: 1.93−3.47) for non-GSK brands, and 1.77% (95% CI: 1.42−2.20) for unknown vaccine brands (Table 4). The ICC was 0.0012 for non-GSK vaccine brands and −0.0006 for unknown brands.

### Cumulative incidence rates of AEIs reported via the EHR or derived from the AERC according to pre-specified clinical category

The total cumulative rates of individual pre-specified AEIs during the follow-up period (weeks 37 to 48) reported for all vaccines are shown in Table 5. To evaluate any potential safety signal with *Fluarix Tetra*, the frequency of individual AEIs observed in the present EPS study was compared with the frequency of the AEIs included in the *Fluarix Tetra* Summary of Product Characteristics (SmPC).^19^ All AEIs except headache occurred at a lower frequency in the present study than stated in the SmPC, and none occurred at a higher frequency (Table 6), confirming that no safety signal was detected for *Fluarix Tetra* in the EPS.

**Table 5.** Cumulative incidence rates of AEIs reported on the EHR or by AERC within the 7 days post-vaccination period by vaccine group according to EMA-specified clinical category (week 37–48).

	*Fluarix Tetra* N = 13,861				Non-GSK N = 2295				Unknown brand N = 3178				All vaccinated N = 19,334			
			95% CI				95% CI				95% CI				95% CI	
AEIs	n	%	LL	UL	n	%	LL	UL	n	%	LL	UL	n	%	LL	UL
Any AEIs	1049	7.60	6.43	8.96	62	2.59	1.93	3.47	54	1.77	1.42	2.20	1165	6.04	5.05	7.22
Fever/pyrexia	133	0.97	0.80	1.18	25	1.20	1.06	1.36	16	0.46	0.26	0.83	174	0.91	0.74	1.12
Local symptoms (i.e. local erythema)	127	0.92	0.57	1.47	0	-	-	-	0	-	-	-	127	0.69	0.40	1.16
Any general non-specific symptoms	202	1.45	1.14	1.84	1	0.09	0.07	0.12	1	0.03	0.00	0.21	204	1.05	0.80	1.38
Drowsiness	72	0.52	0.38	0.70	0	-	-	-	0	-	-	-	72	0.37	0.26	0.52
Fatigue	169	1.20	0.94	1.54	0	-	-	-	1	0.03	0.00	0.21	170	0.87	0.64	1.17
Irritability	17	0.12	0.07	0.20	0	-	-	-	0	-	-	-	17	0.09	0.05	0.14
Malaise	4	0.03	0.01	0.08	1	0.09	0.07	0.12	0	-	-	-	5	0.03	0.01	0.06
Any respiratory/miscellaneous	513	3.66	2.95	4.52	23	0.83	0.68	1.01	13	0.34	0.16	0.71	549	2.73	2.22	3.36
Conjunctivitis	28	0.19	0.10	0.35	2	0.10	0.09	0.12	0	-	-	-	30	0.14	0.07	0.25
Coryza	96	0.64	0.41	1.01	0	-	-	-	0	-	-	-	96	0.44	0.28	0.68
Cough	210	1.53	1.18	2.00	17	0.59	0.48	0.72	13	0.34	0.16	0.71	240	1.22	0.97	1.52
Epistaxis	12	0.09	0.04	0.16	1	0.09	0.07	0.12	0	-	-	-	13	0.07	0.04	0.12
Hoarseness	71	0.51	0.37	0.69	0	-	-	-	0	-	-	-	71	0.36	0.27	0.48
Nasal congestion	276	1.87	1.42	2.45	3	0.17	0.04	0.63	0	-	-	-	279	1.38	0.98	1.94
Oropharyngeal pain	155	1.10	0.83	1.45	2	0.09	0.02	0.36	0	-	-	-	157	0.78	0.59	1.03
Rhinorrhoea	243	1.69	1.32	2.16	2	0.11	0.02	0.68	0	-	-	-	245	1.23	0.90	1.68
Wheezing	63	0.45	0.28	0.73	0	-	-	-	1	0.03	0.01	0.19	64	0.31	0.20	0.47
Any gastrointestinal	154	1.09	0.85	1.39	2	0.10	0.08	0.12	3	0.10	0.03	0.32	159	0.80	0.60	1.08
Decreased appetite	50	0.35	0.24	0.51	1	0.04	0.01	0.32	1	0.03	0.00	0.26	52	0.26	0.17	0.40
Diarrhoea	64	0.46	0.36	0.59	0	-	-	-	2	0.06	0.02	0.26	66	0.34	0.26	0.45
Nausea	71	0.51	0.38	0.67	0	-	-	-	0	-	-	-	71	0.36	0.26	0.50
Vomiting	19	0.14	0.09	0.21	1	0.09	0.07	0.12	0	-	-	-	20	0.10	0.07	0.15
Any sensitivity/anaphylaxis	15	0.10	0.05	0.21	1	0.09	0.07	0.12	0	-	-	-	16	0.08	0.04	0.16
Anaphylactic reactions	0	-	-	-	0	-	-	-	0	-	-	-	0	-	-	-
Facial oedema	1	0.01	0.00	0.05	0	-	-	-	0	-	-	-	1	0.01	0.00	0.03
Hypersensitivity reactions	14	0.09	0.04	0.21	1	0.09	0.07	0.12	0	-	-	-	15	0.08	0.04	0.16
Any rash	47	0.35	0.24	0.50	6	0.20	0.06	0.60	2	0.06	0.02	0.21	55	0.29	0.21	0.40
Generalised rash	31	0.24	0.15	0.37	6	0.20	0.06	0.60	2	0.06	0.02	0.21	39	0.21	0.14	0.32
Rash	16	0.12	0.08	0.16	0	-	-	-	0	-	-	-	16	0.08	0.05	0.13
Any musculoskeletal	331	2.38	1.89	3.01	7	0.12	0.02	0.67	15	0.47	0.23	0.95	353	1.81	1.41	2.32
Arthropathy	79	0.55	0.33	0.92	0	-	-	-	0	-	-	-	79	0.39	0.22	0.68
Muscle aches/myalgia	311	2.25	1.77	2.87	7	0.12	0.02	0.67	15	0.47	0.23	0.95	333	1.72	1.34	2.20
Any neurological	206	1.50	1.20	1.88	6	0.30	0.26	0.35	5	0.19	0.07	0.56	217	1.12	0.88	1.41
Bell’s palsy	0	-	-	-	0	-	-	-	0	-	-	-	0	-	-	-
Guillain-Barre Syndrome	0	-	-	-	0	-	-	-	0	-	-	-	0	-	-	-
Headache	192	1.40	1.12	1.74	6	0.30	0.26	0.35	5	0.19	0.07	0.56	203	1.05	0.83	1.32
Peripheral tremor	24	0.16	0.12	0.22	0	-	-	-	0	-	-	-	24	0.12	0.09	0.16
Seizure/Febrile convulsions	2	0.01	0.00	0.05	0	-	-	-	0	-	-	-	2	0.01	0.00	0.03

N: number of participants vaccinated.

n: number of participants reporting an AEI at least once.

%: percentage of participants reporting an AEI at least once estimated from logistic GEE models adjusted for clustering effect of general practices, with upper and lower limits of the 95% CI based on the robust variance estimate.

AEI: adverse event of interest; AERC: adverse event reporting card; EHR: electronic health record; EMA: European Medicines Agency; GEE: generalized estimating equation; LL: lower limit; UL: upper limit; 95% CI: 95% confidence interval.

**Table 6.** Observed frequency of AEIs for *Fluarix Tetra* in EPS (AERC and EHR data combined) compared with the frequency of solicited events included in the summary of product characteristics.

	Observed frequency in the SmPC				Observed frequency in the EPS % (95% CI)
	Age group 6 months–3 years	Age group 3–6 years	Age group 6–18 years	Age group ≥18 years	All age groups
**General disorders and administration site conditions**					
Fever	Common	Common	Common	Common	Uncommon: 0.97 (0.80, 1.18)
Fatigue	N/A	N/A	Very common	Very common	Common: 1.20 (0.94, 1.54)
Injection site redness	Very common	Very common	Very common	Common	Uncommon: 0.92 (0.57, 1.47)^a^
**Nervous system disorders**					
Headache	N/A	N/A	Common	Common	Common: 1.40 (1.12, 1.74)
Drowsiness	Very common	Common	N/A	Uncommon	Uncommon: 0.52 (0.38, 0.70)
**Psychiatric disorders**					
Irritability	Very common	Very common	N/A	N/A	Uncommon: 0.12 (0.07, 0.20)
**Musculoskeletal disorders**					
Myalgia	N/A	N/A	Very common	Very common	Common: 2.25 (1.77, 2.87)
Arthralgia	N/A	N/A	Common	Common	Uncommon: 0.55 (0.33, 0.92)^b^
**Gastrointestinal disorders**					
Nausea	N/A	N/A	Common	Common	Uncommon: 0.51 (0.38, 0.67)
Vomiting	N/A	N/A	Common	Common	Uncommon: 0.14 (0.09, 0.21)
Diarrhoea	N/A	N/A	Common	Common	Uncommon: 0.46 (0.36, 0.59)
**Metabolism and nutrition disorders**					
Loss of appetite	Very common	Common	N/A	N/A	Uncommon: 0.35 (0.24, 0.51)
**Skin and subcutaneous tissue disorders**					
Rash	N/R	Uncommon	Uncommon	N/A	Uncommon: 0.35 (0.24, 0.50)

Adverse reactions reported for *Fluarix Tetra* in the different age groups are listed according to the following frequency categories per dose in the SmPC^19^: very common: ≥1/10; common: ≥1/100 to <1/10; uncommon: ≥1/1,000 to <1/100; rare: ≥1/10,000 to <1/1,000); very rare: <1/10,000.

^a^In the present study, this event was captured under local erythema; ^b^In the present study, this event was captured under arthropathy.

N/A = Not solicited in this age group.

N/R = Not reported.

AEI: Adverse event of interest; AERC: Adverse event reporting card; EHR: electronic health record; EPS: enhanced passive surveillance; SmPC: Summary of Product Characteristics; 95% CI: 95% confidence interval (Clopper-Pearson exact CI modified for cluster data).

### Hospital admission and deaths

A total of 12 participants were hospitalized within the 7 days post-vaccination period (nine participants vaccinated with *Fluarix Tetra* and three with an unknown vaccine brand). None were considered to be associated with vaccination based on their registered GP’s judgement. There were no deaths among study participants.

## Discussion

The purpose of our study was to fulfil EMA pharmacovigilance requirements for *Fluarix Tetra* using an EPS method that collected data on pre-defined AEIs, using a combination of data routinely collected from EHRs and a reporting card system completed by vaccinees. We demonstrated that this method can be successfully implemented in a primary care setting.

The study identified no safety signal for *Fluarix Tetra*. The pattern of AEI reporting rates appeared similar from week to week. Compared with the *Fluarix Tetra* SmPC,^19^ the reporting rates of individual AEIs were of similar or lower magnitude, and the cumulative end-of-season analysis revealed no unexpected events. Furthermore, none of the hospital admissions in the week following vaccination appeared to have a causal link with vaccination based on the data available at the weekly reviews and follow-up information requested. The overall incidence rate of AEIs for *Fluarix Tetra* was 4.96% (95% CI: 3.92−6.25) reported via AERC only and 7.60% (95% CI: 6.43−8.96) reported via the AERC or through data recorded in clinical consultations. The incidence rate for non-GSK influenza vaccines based on data recorded in clinical consultations was 2.59% (95% CI: 1.93−3.47). The rate of AEIs appeared to peak early in the season and decline over time. The reason for this is unclear, but we speculate that people who attend early in the season for their influenza vaccine are highly organized individuals who may be meticulous in reporting AEIs. However, we have no specific evidence to support this hypothesis.

Just under half (44.7%) of the *Fluarix Tetra* recipients who were issued with an AERC returned it. Two thirds of participants who reported an AEI with *Fluarix Tetra* did so via the AERC and one third at a GP consultation. It appears that the AERC was a useful tool that complemented routine pharmacovigilance, and allowed more comprehensive capture of AEIs experienced. An Australian study also described a trebling of AEI reports with Bacillus Calmette-Guérin vaccine following introduction of an EPS scheme, although the largest increase was observed among children.^20^ The contribution of EPS in monitoring AEIs for other vaccines has been demonstrated in Australia and the Netherlands.^21,22^

Despite the demonstrated added value of combining routinely collected data from the EHR with data from the AERC, we cannot completely rule out potential under-reporting of AEIs in our study. In an Australian post-marketing surveillance study of healthcare professionals in which surveillance was conducted by short message service (SMS), one in eight (13.3%) recipients of influenza vaccine reported an AEI.^23^ A study in the Netherlands that used three repeated web-based questionnaires, with response rates of 91%, 85%, and 78%, found an AEI rate of 38.5%.^24^ A US study has evaluated adverse events using a system in which influenza vaccine recipients were asked to report via internet or telephone on a daily basis for 14 days post-vaccination regardless of whether an event was experienced or not; daily email reminders were sent and non-respondents were followed up by telephone.^25^ The study found that 62% of people reported AEIs, though half were minor. Interestingly, minor AEIs peaked on the second day and more serious effects (e.g. hoarseness or wheezing, unusual weakness, dizziness) peaked on the fifth day post-vaccination.^25^

The current study was sufficiently powered to detect very common (≥1/10), common (≥1/100 to <1/10) and uncommon (≥1/1,000 to <1/100) AEIs that are pre-defined by EMA, but was not designed to detect rare events (>1/10,000 and <1/1000) or very rare events (<1/10,000).^19^ The ICC was low and the generalized estimating equations (GEE) adjusted percentages of AEIs were close to those reported from the unadjusted data, suggesting that the practices selected for this study were relatively similar in their approach to influenza vaccination and AEI reporting. Weekly analysis was more robust as vaccine exposure increased. Collecting data over successive seasons would provide information about year-on-year variation in rates of AEIs and may make it easier to detect any safety signal. Longitudinal data would be particularly helpful at the start of the season when it is hard to tell if sporadic reports represent any type of signal.

Our data are primarily applicable to adult vaccine recipients. In the UK, the live attenuated influenza vaccine nasal spray is used preferentially for children, and thus very few children received *Fluarix Tetra* and were issued with an AERC. Therefore, the data from our study do not fully represent all influenza vaccine recipients in England. A limitation of our study is that pneumococcus and shingles vaccines are commonly administered in UK primary care on the same day as the influenza vaccine, potentially leading to difficulties in distinguishing between events associated with one vaccine versus another. Co-administration of other vaccines during the study was expected to be limited, and therefore documentation of co-administration was not part of the study procedures. A high proportion of ethnicity data was missing. We could improve our EPS method by capturing on the EHR when a returned AERC indicated no AEI. Other options that could have been considered include using reminders to try to boost the return rate of the AERCs and offering an SMS or web-based system to report AEIs rather than a card-based system.

## Conclusions

Our EPS method combined routinely-collected data from the EHR with data from an AERC in people vaccinated with *Fluarix Tetra* to maximize the likelihood of capturing any AEI experienced. We succeeded in enrolling a substantial number of vaccinated people, exceeding the minimum sample size recommended by EMA (n = 1000),^10^ as well as the anticipated number used in our sample size assumptions.^17^ We demonstrated that our EPS method can be successfully implemented in a primary care setting. No safety signal for *Fluarix Tetra* was identified. Our EPS method combining routine data from the EHR with an AERC appears to be a useful way to improve capture of AEIs compared with classical passive surveillance alone. In conclusion, the study identified no safety signal during or at the end of the study that would impact public health or alter the benefit-risk profile of *Fluarix Tetra*. The study supports and confirms the safety profile of GSK’s *Fluarix Tetra* vaccines.

## Methods

The study methods have been published previously in detail^17^ and are summarized below. In summary, our EPS method was a combination of near-real-time passive surveillance of EHR systems, enhanced by use of an AERC in participants vaccinated with *Fluarix Tetra*; the method followed EMA guidance.^10^ The study received a favourable ethics committee approval: Integrated Research Application System (IRAS) project ID: 211560; Research Ethics Committee (REC) reference: 16/NE/0271. All data were pseudonymized, as described previously.^17^ Interim and final analyses were performed. Interim safety reports were assessed on a weekly basis by GSK’s safety core team to identify any potential safety concerns. The content and outcome of those investigations were communicated to the University of Surrey who could contact the participating sites for further insights as appropriate. We have previously reported an interim analysis of this study, where the AERCs were described as “orange cards” but refer to the same tool^18^; the present paper reports the final analysis.

### Setting and population

The sample size calculation has been reported previously.^17^ Briefly, assuming that influenza vaccination uptake in the 2016−2017 season would be similar to the previous season, we estimated that approximately 70,000 individuals would be required to enroll approximately 5000 vaccinees. As the average practice size is 7034, we recruited 10 practices.

We enrolled 10 general practices that used GSK’s *Fluarix Tetra* as their principal brand of influenza vaccine, although other brands were also used. These practices were already part of the Royal College for General Practitioners Research and Surveillance Centre network. We picked the practices based on their principal vaccine brand, their representativeness of the general population and their willingness to participate. The practices were spread across England: three practices each in the North, Midlands and East, and South NHS regions, with the tenth practice in London, and encompassed conurbation, urban and rural areas. The practices used different EHR systems; seven used EMIS Webb, one used TPP SystmOne and one used INPS Vision. The process was the same for all clinical systems. Reimbursement to the practices followed National Institute of Health Research guidelines for industry sponsored studies. Before the start of the study, each participating GP was provided with induction training which covered the scope of the study and the method of achieving standardized encoding of the events reported. In addition, GPs were instructed on the need to prime vaccinees about the study and the importance of reporting any post-vaccination events.

The study vaccination period covered 1 September to 30 November 2016 and the follow-up period captured AEIs from week 37 to week 48. All individuals in an enrolled practice who received a seasonal influenza vaccination were eligible for the study providing they had a valid and pseudonymized NHS number, date of birth and gender recorded in the EHR. Individuals who received their vaccination outside of the practice (e.g. at a pharmacy) were included in the analysis. Any individual who was vaccinated with *Fluarix Tetra* outside the general practice did not receive an AERC. Individuals who had explicitly opted out of data-sharing were excluded. The study had no influence on which individuals were vaccinated or with which vaccine, although it was anticipated that national guidance would be followed.^26^ These guidelines recommend that the following groups be vaccinated: people ≥65 years of age; people with a chronic medical condition; pregnant women; children 2, 3 or 4 years of age; and other individuals considered at risk or a close contact of people at risk.^26^ However, any individual outside of the recommended groups could choose to be vaccinated.

### Data collection

We utilized the database and weekly reporting system developed in 2015 for the Royal College of General Practitioners Research and Surveillance Centre, the primary care national surveillance system.^27,28^ We used two data sources: (1) practice EHR data (passive surveillance component) and (2) AERC completed by participants receiving *Fluarix Tetra* (enhanced component). The AERCs (the ‘orange card’) have been described previously.^17,18^ The cards were handed out at the routine vaccination and were returned either in person or by mail, ideally within 7 days post-vaccination, but no longer than 14 days. We performed a weekly pseudonymized data extract utilizing both data sources. Cross-sectional weekly reports were produced and published online.^29^ A summary of the results is available on ClinicalTrials.gov (NCT02893878).

#### Vaccine exposure

The total number of people vaccinated was recorded and categorized as (1) vaccinated using GSK’s *Fluarix Tetra*; (2) vaccinated with another specified brand of influenza vaccine (recorded as ‘Non-GSK’); (3) vaccinated with an unknown brand of influenza vaccine. We reported the characteristics of the population exposed to influenza vaccine including age category at the time of immunization (6 months to 5 years, 6−12 years, 13−17 years, 18−65 years, >65 years); gender; ethnicity using UK Office of National Statistics categories and an ontological approach to maximize data capture^30^; and a measure of socioeconomic status, the Index of Multiple Deprivation (IMD).^31^ In addition, we reported vaccine exposure according to high-risk group specified by national guidance if applicable, and added a category “not at risk” where no obvious risk factor was recorded.

Vaccine exposure data were either reported using a vaccine administration code, or through recording of vaccine brand and batch number. However, the vaccine brand was not consistently recorded when the vaccine was administered in a pharmacy, because recording of the brand is not required by the system that provides data about pharmacy administration.^32^ We reported vaccine exposure data when either the date of administration or the batch number was available; data were considered complete if both administration date and batch number were available.

#### Adverse events

The AERC consisted of a structured list of pre-specified AEIs recommended by the EMA,^10^ with the option for participants to record that no AEI or any adverse event occurred. Events were grouped into body systems: respiratory conditions (conjunctivitis, coryza, cough, epistaxis, hoarseness, nasal congestion, oropharyngeal pain, rhinorrhea, wheezing); musculoskeletal conditions (arthropathy, muscle aches/myalgia); neurologic conditions (Bell’s palsy, Guillain-Barre syndrome, headache, peripheral tremor, seizure/febrile convulsions); gastrointestinal conditions (decreased appetite, diarrhea, nausea, vomiting); general symptoms (drowsiness, fatigue, irritability, malaise); sensitivity/anaphylaxis (anaphylactic reactions, facial edema, hypersensitivity reactions); fever/pyrexia including actual temperatures recorded when available; local erythema; and rash.

Adverse events were recorded for 7 days post-vaccination. The practice recorded all returned AERCs. Any adverse events recorded on the AERC were entered on the participant’s EHR, but if no adverse event was recorded, this was not captured on the EHR. AEIs reported to the practice outside of the AERC were recorded on the EHR.

#### Hospital admissions and deaths

Hospital admissions were identified via our data extracts using a set of read codes. These varied in specificity and may not have given the reason for the admission. Therefore, the practice at which the participant was registered was contacted within a week from admission to determine the reason for the admission and any suspected causal link with vaccination in the professional judgement of the practice. Each practice was also required to provide the reason for any deaths and any suspected causal link with the vaccine. If a causal link was suspected, practices were encouraged to use the national reporting system “Yellow Card Scheme” in parallel to the reporting scheme described in this paper; in addition, if the event occurred after vaccination with *Fluarix Tetra*, the practice was required to report the event to GSK in parallel.

### Study endpoints

The primary endpoint was the occurrence of AEIs within 7 days post-vaccination reported with the AERC in recipients of *Fluarix Tetra*. Data were stratified by age category (6 months−5 years, 6−12 years, 13−17 years, 18−65 years, >65 years). The secondary endpoint was the occurrence of AEIs within 7 days post-vaccination using data from the EHR (which includes data from the AERC), with data stratified by vaccine brand (*Fluarix Tetra*, non-GSK or unknown). Influenza vaccine exposure and return rate of the AERCs were analyzed as tertiary endpoints. As part of EMA pharmacovigilance requirements, the study also evaluated any potential safety signal with *Fluarix Tetra* by comparing the observed frequency of individual AEIs versus the frequency of solicited AEIs included in the *Fluarix Tetra* SmPC.^19^ The occurrence of any unexpected event or an increase in frequency versus the SmPC could indicate a potential safety signal.

### Statistical analysis

Weekly data were reported in the weekly vaccinated safety cohort which included all registered individuals who were eligible for the study and vaccinated during the week before the week of interest (reaching up to 7 days of follow-up post-vaccination during the week of interest). Cumulative data were reported in the cumulative vaccinated safety cohort which included all registered individuals who were eligible for the study and vaccinated at any point from study start-up to the week before the week of interest (i.e. cumulatively since the beginning of the study).

The number and proportion of individuals who received an influenza vaccine in the 10 general practices were calculated. We used descriptive statistics to report demographic characteristics. Recruitment into the study by GP practices may have created a clustering effect, because participants in the same practice are more likely to receive similar treatment for a given condition, and are likely to share similarities including geography, socioeconomic status, ethnic background, or age by virtue of the area in which they live. In the same way, GP practices that have chosen to work together are likely to share similarities. Similarities (or homogeneity) between participants in clusters reduce the variability of their responses, compared with that expected from a random sample. This clustering effect thus decreases the precision of the estimated rates (i.e. leading to a wider confidence interval around the estimated rates).

The incidence rates of AEI, their 95% CI and ICCs accounting for clustering effect were estimated from the logistic GEE models adjusted for clustering effect of GP practices with a robust variance estimate.^33^ The exact CI for a proportion within a group, not accounting for clustering effect, was calculated using the method described by Clopper & Pearson.^34^

For the weekly incidence rate of AEIs, the denominator was the number of participants in the weekly vaccinated safety cohort for the week of interest and the numerator was the corresponding number who reported an AEI within 7 days following vaccination with the seasonal influenza vaccine. For the cumulative incidence rate, the denominator was the number of participants in the cumulative vaccinated safety cohort up to the week of interest and the numerator was the corresponding number who reported an AEI within 7 days following vaccination with the seasonal influenza vaccine.

## Trademark statement

*Fluarix Tetra* is a trademark of the GSK group of companies.
