# Supplementary material for: Enhanced passive surveillance of influenza vaccination in England, 2016−2017– an observational study using an adverse events reporting card
Source: Hum Vaccin Immunother. 2019 Mar 20;15(5):1048–59. doi: 10.1080/21645515.2019.1565258 (PMC6605873; doi:10.1080/21645515.2019.1565258)
Supplement: Supplemental Material [file khvi-15-05-1565258-s001.docx]

# Supplement

**Supplement Table 1. Completeness of seasonal influenza vaccination data in the EHR**

|  |  | ***Fluarix Tetra***  **N=13,861** | | **Non-GSK**  **N=2295** | | **Unknown brand**  **N=3178** | | **All vaccinated**  **N=19,334** | |
| --- | --- | --- | --- | --- | --- | --- | --- | --- | --- |
| **Characteristics** | **Categories** | **n** | **%** | **n** | **%** | **n** | **%** | **n** | **%** |
| Complete data* on seasonal influenza vaccine in EHR | Yes | 13,807 | 99.6 | 2277 | 99.2 | 109 | 3.4 | 16,193 | 83.8 |
|  | No | 54 | 0.4 | 18 | 0.8 | 3069 | 96.6 | 3141 | 16.2 |
| Seasonal influenza vaccine administration date | Complete date in EHR | 13,861 | 100 | 2295 | 100 | 3178 | 100 | 19,334 | 100 |
|  | Incomplete date in EHR | 0 | 0.0 | 0 | 0.0 | 0 | 0.0 | 0 | 0.0 |
| Seasonal influenza vaccine batch number in EHR | Yes | 13,807 | 99.6 | 2277 | 99.2 | 109 | 3.4 | 16,193 | 83.8 |
|  | No | 54 | 0.4 | 18 | 0.8 | 3069 | 96.6 | 3141 | 16.2 |

*Administration date and batch number

N = number of participants

n/% = number / percentage of participants in a given category

EHR: electronic health record

**Supplement table 2. Weekly and cumulative weekly incidence rates of any AEI reported via AERC within 7 days post vaccination in participants receiving *Fluarix Tetra***

|  | **Weekly incidence** | | | | | **Cumulative weekly incidence** | | | | |
| --- | --- | --- | --- | --- | --- | --- | --- | --- | --- | --- |
|  | **N** | **n** | **%** | **95% CI** | | **N** | **n** | **%** | **95% CI** | |
| **Week** |  |  |  | **LL** | **UL** |  |  |  | **LL** | **UL** |
| 37 | 6 | 0 | - | - | - | 6 | 0 | - | - | - |
| 38 | 1269 | 67 | 4.36 | 2.54 | 7.39 | 1275 | 67 | 4.30 | 2.49 | 7.33 |
| 39 | 2626 | 169 | 6.21 | 5.01 | 7.66 | 3901 | 236 | 5.79 | 4.65 | 7.18 |
| 40 | 2146 | 164 | 7.48 | 6.00 | 9.30 | 6047 | 400 | 6.48 | 5.31 | 7.90 |
| 41 | 2458 | 112 | 4.75 | 3.57 | 6.28 | 8505 | 512 | 5.91 | 4.72 | 7.38 |
| 42 | 1362 | 85 | 7.46 | 7.34 | 7.59 | 9867 | 597 | 5.85 | 4.69 | 7.26 |
| 43 | 738 | 29 | 4.16 | 2.58 | 6.65 | 10,605 | 626 | 5.76 | 4.66 | 7.10 |
| 44 | 769 | 31 | 3.32 | 1.78 | 6.11 | 11,374 | 657 | 5.61 | 4.52 | 6.94 |
| 45 | 704 | 28 | 3.94 | 2.10 | 7.26 | 12,078 | 685 | 5.49 | 4.40 | 6.84 |
| 46 | 945 | 13 | 1.69 | 0.87 | 3.23 | 13,023 | 698 | 5.25 | 4.17 | 6.60 |
| 47 | 474 | 3 | 0.74 | 0.18 | 3.10 | 13,497 | 701 | 5.08 | 4.03 | 6.40 |
| 48 | 364 | 2 | 0.67 | 0.10 | 4.37 | 13,861 | 703 | 4.96 | 3.92 | 6.25 |

N: number of participants vaccinated with *Fluarix Tetra*

n: number of participants reporting an AEI at least once on the AERC

%: percentage of participants reporting an AEI at least once on the AERC estimated from logistic GEE models adjusted for clustering effect of general practices, with upper and lower limits of the 95% CI based on the robust variance estimate

AEI: adverse event of interest; AERC: adverse event reporting card; CI: 95% confidence interval; GEE: generalized estimating equation; LL: lower limit; UL: upper limit
